# Volatile Variation of *Theobroma cacao* Malvaceae L. Beans Cultivated in Taiwan Affected by Processing via Fermentation and Roasting

**DOI:** 10.3390/molecules27103058

**Published:** 2022-05-10

**Authors:** Li-Yun Lin, Kwei-Fan Chen, Lin-Ling Changchien, Kuan-Chou Chen, Robert Y. Peng

**Affiliations:** 1 Department of Food and Applied Technology, Hungkuang University, No. 1018, Sec. 6, Taiwan Boulevard, Shalu District, Taichung City 43302, Taiwan; lylin@hk.edu.tw (L.-Y.L.); kfchen@hk.edu.tw (K.-F.C.); 2 Department of Physical Therapy, Hungkuang University, No. 1018, Sec. 6, Taiwan Boulevard, Shalu District, Taichung City 43302, Taiwan; llchangchien@hk.edu.tw; 3 Graduate Institute of Clinical Medicine, College of Medicine, Taipei Medical University, No. 250, Wu-Xin St., Taipei 11031, Taiwan; robertpeng120@gmail.com; 4 Department of Urology, Taipei Medical University Shuang-Ho Hospital, 250, Wu-Xin St., Xin-Yi District, Taipei 11031, Taiwan; 5 Research Institute of Biotechnology, School of Health Care, Hungkuang University, 1018, Sec. 6, Taiwan Boulevard, Shalu District, Taichung City 43302, Taiwan

**Keywords:** cocoa bean flavors, fermentation, roasting, volatile changes, triacylglycerols, fats and fatty acids, amino acids, pyrazine and esters

## Abstract

After being harvested, cacao beans are usually subjected to very complex processes in order to improve their chemical and physical characteristics, like tastefulness with chocolate characteristic flavors. The traditional process consists of three major processing stages: fermentation, drying, and roasting, while most of the fermentation is carried out by an on-farm in-box process. In Taiwan, we have two major cocoa beans, the red and the yellow. We proposed that the major factor affecting the variation in tastes and colors in the finished cocoa might be the difference between cultivars. To uncover this, we examined the effect of the three major processes including fermentation, drying and roasting on these two cocoa beans. Results indicated that the two cultivars really behaved differently (despite before or after processing with fermentation, drying, and roasting) with respect to the patterns of fatty acids (palmitic, stearic, oleic, and arachidonic); triacylglycerols:1,2,3-trioleoyl-glycerol (OOO); 1-stearoyl-2,3-oleoyl-glycerol (SOO); 1-stearoyl-sn-2-oleoyl-3-arachidoyl- glycerol (SOA); 1,3-distearyol-sn-2-oleoyl-glycerol (SOS); organic acids (citric, tartaric, acetic, and malic); soluble sugars (glucose and fructose); amino acids; total phenolics; total flavonoids; and volatiles. Our findings suggest that to choose specific processing conditions for each specific cocoa genotype is the crucial point of processing cocoa with consistent taste and color.

## 1. Introduction

Cocoa, a significant agricultural commodity and a cash product of great economic importance in the world, is utilized as the crucial raw material for chocolate and cocoa powder manufacturing [1]. Specifically, the term ‘cacao’ indicates the raw cacao beans or trees, while the term ‘cocoa’ denotes the processed product cocoa or chocolates, however, the two terms have been used interchangeably in many countries, in particular Brazil and Spain [1]. The genus *Theobroma* contains 22 species classified into six sections, most native to the upper Amazon region in South America [2], whereas only *T. cacao* L. and *T. grandiflorum* (cupuassu) are explored commercially on a large scale. *Forastero* genotypes are traditionally cultivated in Brazil and West African countries, and represent most of the commercial production of cocoa [3]. However, cacao trees, like most tropical plants, possess fertility of ‘self-incompatibility’, making genomic classification difficult [4]. Due to their bitter and astringent flavor, moreover, the unpalatable and unpleasant taste, raw cocoa beans are inedible and need to be cured and transformed into good-tasting and full-flavored cocoa and chocolates [4].

After harvested, the cacao beans are usually subjected to very complex processes in order to improve their chemical and physical characteristics, like mouthfeel of chocolate characteristic flavors [5,6]. The traditional process consists of three major processing stages: fermentation, drying, and roasting [1,7]. Curing involves fermentation of the fresh cacao bean pulp mass, followed by drying of the fermented cocoa beans and subsequent roasting of the fermented dry cacao beans. Fermentation plays a significant role in determining the composition and flavor of chocolate and other cocoa-based products, and it hence lies at the top of the entire chocolate-making process [4]. In addition, fermentation renders the beans more easily separated. The next stage is drying to reduce the moisture content to 6–8% to inhibit the fungal growth. The final stage involves a serial treatment including alkalinization, refining, roasting and grinding, in which roasting is the major process. The cocoa liquor obtained is mixed well and press filtered to separate the cocoa butter and the cake, the latter was desiccated at 50 °C, pulverized and cycloned to collect the cocoa powder [7,8], which is the main raw material for making chocolates.

Worth noting, most of the cocoa bean fermentations are likely carried out by a regional on-farm process, depending on the cocoa-producing region, and usually lasts for about 2–10 days, thus leading to end-products of variable quality [4]. An area of potential concern is the contamination from fungal toxins. Although the overall mean levels are low, the concentration range can vary from 0.07 to 7.8 ng/g ochratoxin A (OTA) (2-year survey), and not-detected to −3.52 ng/g total aflatoxins (1-year survey) in cocoa powder [9]. In addition, recently consumers have an advanced demand toward products with differentiated profiles of aroma and flavor [10] (e.g., fruity, floral, nutty, and herbal profiles among others) [11,12,13], as well as a presence of bioactive compounds, such as antioxidants [14,15,16,17].

Several studies have well established that the processing of cocoa beans through means such as fermentation, drying, alkalization and roasting would cause considerable changes in the chemical composition of the final product such as cocoa powder or chocolate [18,19,20,21]. In Taiwan, we have several cultivars or “polymorphisms”, among which the majority are “red” and “yellow” cocoa beans. We propose that the defined processing conditions like fermentation, drying and roasting may play major roles to exert differential influence on different cultivars, which in turn will affect the final taste and flavor characteristics of chocolates, in order to extend such variation information to the chocolate manufacturers, the compositional changes affected via fermentation to roasting processes were examined.

## 2. Results and Discussion

### 2.1. Proximate Analysis

All proximate compositions decreased due to different treatment starting from fermentation (f), drying (d) to roasting (r). fRC (f: fermented, RC: red cocoa) showed proximal compositions as (in % *w*/*w*): moisture (9.31 ± 0.16), crude ash (3.24 ± 000), crude protein (11.51 ± 0.60), crude fat (41.70 ± 0.50), and carbohydrate (34.24), while fYC (f: fermented, YC: yellow cocoa) showed rather comparable results (Table 1). Similar trend was observed for dRC vs. dYC (d: desiccated), and rRC vs. rYC (r: roasted). Apparently, the values depended on the relative moisture content (Table 1). Fresh cocoa beans contain about 32–39% water, 30–32% fat, 10–15% protein, 5–6% polyphenols, 4–6% pentosans, 2–3% cellulose, 2–3% sucrose, 1–2% theobromine, 1% acids and less than 1% caffeine [22,23], moreover, it is also a rich source of mineral components [22,23]. Literature elsewhere reported that based on the moisture content of 3.64 %(*w*/*w*), the contents of crude protein, crude fat, and crude ash of the cacao beans were 14.37, 55.79, and 6.63 %(*w*/*w*), respectively [1]. The crude fat content of Taiwan cocoa beans was still less than the cited. An implication has evidenced the inferior quality of Taiwan cocoa beans, which might be affected by many factors, the genetic factors, and the cocoa’s vulnerability to climate changes [24], which remains an interesting avenue for our future work. For storage and transport, it is advisable to keep the moisture content below 8%, otherwise mold growth is possible [25,26,27,28]. For preservation, the desiccated beans are suggested to be oven roasted at 140 °C until the moisture content dropped to <5%.

### 2.2. Variation of Fatty Acid Pattern

Four fatty acids were detected, i.e., palmitic (16:0), stearic (18:0), oleic (18:1), and arachidic (20:0). In the fermented red cacao beans (fRC), the pattern showed 0.75 ± 0.01, 1.06 ± 0.04, 0.60 ± 0.03, and 0.12 ± 0.03 g/100 g, while correspondingly lower contents were observed in the fermented yellow cacao beans (fYC), giving 0.57 ± 0.04, 0.70 ± 0.03, 0.57 ± 0.01 and 0.08 ± 0.03 g/100 g. All thee contents seemed to increase due to drying, while a great part was lost by roasting. In the roasted red cacao beans (rRC), the pattern changed to 0.75 ± 0.01, 0.93 ± 0.01, 0.72 ± 0.03 g/100 g, and n.d. (not detected), contrasting with those data below 0.25 ± 0.04 g/100 g in the roasted yellow cacao beans (rYC) (Table 2). The percentage loss of these fatty acids from the drying stage to roasting was found larger in dRC than dYC, implicating the existence of different mass transferring microtexture and compartmentation.

### 2.3. Variation of Triacylglyceride Pattern

The main TAGs in cocoa butters are POS (1-palmitoyl-2-oleoyl-3-stearoyl-glycerol), SOS (1,3-distearoyl-sn-2-oleoyl-glycerol), and POP (1,3-dipalmitoyl-sn-2-oleoyl-glycerol), constituting over 97.59% of total TAGs in FRC and 96.84% in FYC (Table 3). Other minor constituents were SOO (1-stearoyl-2,3-oleoyl-glycerol), OOO (1,2,3–trioleoyl-glycerol), and SOA (1-stearoyl-sn-2-oleoyl-3-arachidoyl-glycerol) (Table 3). After roasted, these level changed to POS 55.43 ± 0.13% SOS 22.81 ± 0.05% and POP 19.27 ± 0.02% (Table 3), and those of rYC correspondingly changed to POS 53.01 ± 0.09% SOS 27.21 ± 0.06% and POP 16.18 ± 0.02% (Table 3). The presence of low-melting TAGs (POO and/or SOO) in their TAG composition and their mixing behavior with SOS could be a reason for the observed decrease in crystallization temperature [29]. Obviously, such a difference in triacylglycerol profile could affect the escaping tendency of triacylglycerols during roasting. The contents of SOO and SOA in the roasted cacao beans decreased in rRC, but increased in rYC (Table 3), again evidencing the effect of different microtexture and compartmentation for mass transfer.

### 2.4. Variation of Organic Acid Pattern

Citric, tartaric, succinic, acetic, and malic acids were detected in both cacao beans. Interestingly, citric and succinic acids were not found in fRC, also the levels of acetic and malic acids were very low compared to fYC (Table 4). Literature indicated that by the early intervention of indigenous yeasts and lactic bacilli, citric acid was exhausted within 36 h, whereas acetic acid reached a peak value at 3.5 days [4]. The appearance of elevated citric acid concentration in dRC (177.63 ± 0.18 mg/100 g) suggested the consistently ongoing post fermentation during the drying process before roasted (Table 4). While the high citric acid (335.56 ± 0.19), succinic acid (36.42.48 ± 0.08), acetic acid (720.48 ± 0.08) and malic acid (129.32 ± 0.10 mg/100 g) contents present in the rYC could be due to the late stage fermentation during the period of drying to immediately before roasting (Table 4).

Study of the microbial community dynamics during the fermentation for cacao pulps showed that indigenous yeast appeared in the first phase during 12–36 h [4,30]. In the second phase, the indigenous lactic acid bacilli appeared in the early beginning, reaching a peak at 36–96 h, then declined slightly until day 6. The third phase is dominated by the indigenous acetic acid bacteria at day 3 and reached a peak at 3.5 days [4,30]. During this last phase, an exothermic reaction (alcohol oxidized into acetic acid) occurs, responsible for a temperature rise up to 50 °C or higher. Yeasts also produce organic acids such as acetic and succinic acids [4,30]. Obviously, successful cocoa bean fermentation requires a well compromised succession of these particular microbial activities [4].

### 2.5. Variation of Soluble Sugar Pattern

Contents of sugars and enzymic breakdown of polysaccharides form an important source of precursors [31,32,33,34]. The profile of reducing sugars developed during each stage of the postharvest of cocoa beans is an important quality index [34]. Fresh unfermented mature cocoa beans exhibit relatively higher concentration of sucrose 1.58 g/100 g [35] and trace amounts of fructose, sorbose, mannitol and inositol at zero time of fermentation [35,36]. We found only the presence of glucose (0.735 and 0.404 g/100 g in fRC and dRC, respectively) and fructose (1.480 and 1.483 g/100 g in fRC and fYC, respectively) (Table 5), indicating the hydrolysis of sucrose occurred rapidly during the fermentation process. Consistent with [34], who reported the comparable contents of glucose and fructose to be 0.66 and 1.46 g/100 g, respectively.

### 2.6. Variation of Amino Acid Pattern

The unfermented cocoa beans in fact contain a very low amount of free amino acids [37]. The total amino acid content could increase up to about 150–200% during the fermentation [36,37,38], as shown in Table 6. The protein breakdown is the source of many compounds involved in food sensory properties. Cocoa fermentation is crucial not only for the primary production of important flavor volatiles, but also for the formation of many nonvolatile chemicals, the so-called cocoa flavor precursor [39]. Amino acids formed from the protein hydrolysis mainly proceed through the Ehrlich Neubauer pathway, yielding keto acids, amines, aldehydes, branched chain alcohols and aromatic acids [40]. Some aldehyde can also be produced through anabolic pathway during the amino acid synthesis [41].

During roasting, these amino acids react with glucose via the Maillard reaction to produce characteristic aroma compounds, contributing to a variety of flavor and color to the final food [42].

The early stage of the Maillard reaction involved the sugar-amine condensation and the Amadori rearrangement. The intermediate stage covers a span of sugar breakdown or dehydration, and Strecker degradation. The final stage of the Maillard reaction can be contributed by formations of pyrazines, pyrroles, pyridines, sulfur compounds, and Aldol condensation [43]. Different amino acids such as leucine, isoleucine, and D-glucose produce different flavors at different stage of Maillard reaction [43]. Tyrosine, serine, and alanine produce caramel aroma under the same conditions [44]. L-proline and L-hydroxyproline produces corny, cereal-, toasty- and cracker-like aroma [42], resulting in the typical aromatic compounds of chocolate [42,45]. Evidently, a large proportion of proline and hydroxyproline was converted via such a pathway, leading to the entire exhaustion of proline contents in dRC, rRC, fYC, dYC and rYC (Table 6).

### 2.7. Variation of Total Phenolics and Flavonoids

The contents of total phenolic (TPC expressed in GAE, mg/mL) and total flavonoid (TFC expressed in QE mg/mL) were found to be highest in the fermented beans. The ethanolic extraction showed 2–3 fold higher extractability than the water extraction (Table 7).

Most of which decreased via drying, and to the least after being roasted. Roasting destroyed most parts of TPC (Table 7). In the aqueous extracts, the contents of TPC in fRC, dRC, and rRC were 8.94 ± 0.09; 4.74 ± 0.02, and 4.74 ± 0.02; and those in fYC, dYC, and rYC were 6.52 ± 0.06; 3.85 ± 0.05, and 2.54 ± 0.11, respectively (Table 7). Compared to the ethanoic extracts, the TPC were: fRC (16.35 ± 0.04), dRC (12.65 ± 0.08), and rRC (8.65 ± 0.06); fYC (20.15 ± 0.11), dYC (18.61 ± 0.23), and rYC (10.85 ± 0.07), respectively (Table 7). Similar trend was found for TFC, and more worth noting, TFC was more thermal stable than TPC (Table 7). Factors controlling the extractability involve the kinds of solvent, the concentration of solvent, pH, temperature, irradiation time, the solid-to-solvent ratio [21,46], as well as the mesh size of the material particles [46]. In processing, fermentation and drying of the cocoa beans lead to oxidative degradation of polyphenols as a result of contact with the oxidative enzymes, polyphenol oxidase (PPO) and peroxidase [18,19]. However, accumulating studies have demonstrated that the process of determining the polyphenols content in chocolates is rather complex, it is not clear which cocoa beans are the best source of polyphenols [47].

### 2.8. Variation of Antioxidative Capability

The antioxidative ability expressed as either DPPH or ABTS^+^ scavenging capability in trolox equivalent (mg/mL) was rather strong for all processed coco beans (Table 8), however its antioxidative capability was not in parallel with TPC or TFC (Table 7). During cocoa processing, the naturally occurring antioxidants (flavonoids) are lost, while others, such as Maillard reaction products, are formed. The final content of antioxidant compounds and its related antioxidant activity is in fact a function of several variables, some related to processing and formulation, while some related to the newly formed material [48], as well cited, the inherent polyphenols may also react with proteins, free amino acids, and mono- or polysaccharides during roasting to form new compounds with antioxidant activities [18,19].

### 2.9. Volatile Compounds

The most important processes, involving most of the reactions important for development of the proper chocolate flavor, are fermentation, drying and roasting of cocoa beans, and chocolate conching [20]. Subject literature reports different temperature and time ranges for roasting of cocoa beans, but most often mentioned are the temperature of 130–150 °C and time of 15–45 min [49].

The flavor precursors formed during cocoa fermentation are converted into two main classes of flavor-active compounds, aldehydes and pyrazines during roasting, thus completing the spectrum of chemical compounds that comprise cocoa flavor [39,50,51,52]. Roasting reduces contents of undesirable components, produces chocolate-specific aroma and flavor, and decontaminates the cocoa beans [50,51,52]. About 600 several compounds (alcohols, esters, aldehydes, ketones, carboxylic acids, and pyrazines) have been recognized as odor-active compounds [1]. Some fine volatiles were assumed to be pulp-derived (e.g., linalool, β-myrcene, 2-heptylacetate) or intrinsic to the bean (e.g., 2-heptanol, 2-heptanone, 2-pentanol), while others were generated during fermentation by microbial synthesis (e.g., 2-phenylethanol, isoamyl alcohol) [53] (Table 9 and Table 10). In this study, we only recognized the presence of 81 volatile compounds (Table 9 and Table 10), which included 25 alcohols, 9 aldehydes, 13 ketones, 4 acids, 8 terpenes, 15 pyrazines, and 7 others (Table 9 and Table 10). According to much of previous studies, these volatiles could represent the most effective compounds in creating the cocoa products’ flavor [1].

Pyrazines and esters were two major groups of cocoa aroma compounds formed during roasting via Maillard reaction [1]. Tetramethylpyrazine and trimethylpyrazine are the most important pyrazines. Trimethylpyrazine exhibits nutty, grassy, and constant cocoa notes, while tetramethylpyrazine imparts the gravy properties to the cocoa flavor [54]. We detected trimethylpyrazine (μg/g) in fRC (6.90), dRC (0.40), rRC (3.25), fYC (0.40), dYC (n.d.), and rYC (11.89); and other trisubstituted pyrazines like 2-ethyl-3,5-dimethylpyrazine in rRC (6.36), 2-ethyl-3,6-dimethylpyrazine in rYC (11.50), 5-ethyl-2,3-dimethylpyrazine (1.80) and 3,5-diethyl-2-methylpyrazine in rRC (1.12) (Table 10). The tetramethyl pyrazine was detected in fRC (μg/g) (30.15), dRC (1.65), and rRC (3.87), but not found in all treated yellow varieties (Table 10); while the tetrasubstituted pyrazine 2,3,5-trimethyl-6-ethylpyrazine was uniquely found in the roasted rRC (1.33) and rYC (3.10) (μg/g) (Table 10), inconsistent with [55]. In contrast, literature reported the occurrence of only one trimethylpyrazine (2,3,5-trimetyhylpyrazine) and one tetramethylpyrazine (2,3,5,6-tetramethylpyrazine) [1]. Such a deviation could arise due to the difference of different cultivars and roasting temperatures (our 140 °C vs. 115–120 °C).

Esters, mostly acetates, playing a fruity flavor and acting as the generic aroma components [56], are the second most significant flavors after pyrazines [54,57]. A total of 17 esters were found (Table 9 and Table 10). Three major esters were isoamylacetate, 2-phenylethylacetae, and isobutyl benzoate (Table 9), consistent with Mohamadi [1]. The next high levels included dimethyl sulfuroate, ethyl hexanoate, 1-methylhexylacetate, and isoamyl butyrate (Table 10). 2-Phenylethylacetate displays flowery and honey notes [58]. Remarkably, fRC showed extraordinarily high level of isoamylacetate (101.26 μg/g) and 2-phenylethylacetate (22.11 μg/g), which were reduced after roasted in rRC (9.27 μg/g), whereas these two esters increased in rYC after roasting (Table 10). High temperature roasting negatively affected the content of esters [54], controversially, Jinap reported that the highest esters formation was obtained in nib roasted at higher temperatures (160–170 °C) with a shorter time of 5–15 min [58]. To compare, in this study the cocoa beans were roasted at medium high temperature (140 °C). A great amount of aldehydes as well as ketones is desirable and critical for development of cocoa characteristics [1,13]. Usually, aldehydes are derived from Strecker degradation of free amino acids during roasting and are vital for the development of the cocoa aroma [59]. Some aldehyde can also be produced through anabolic pathway during the amino acid synthesis [41].

fRC and rRC exhibited rather high level of 2-methylbutanal (13.97 and 89.15 μg/g, respectively), which is a good flavor displaying musty, chocolate, nutty with malty and fermented nuances odor, and tastes like musty, furfural, rummy, and caramel and fruity undernotes. Curiously, it was totally unseen in the yellow cultivar (Table 10). Conversely, benzaldehyde was entirely absent in the red cacao beans (Table 10). Importantly, the appearance of 2-phenyl-2-butenal in rRC and rYC was relatively high. Benzaldehyde and phenylacetaldehyde (benzene acetaldehyde) display honey and floral perception, while 2-phenyl-2-butenal exhibits sweet honey like chocolate sensory odor [13]. High temperatures and a longer roasting period reduce the quantity of aldehydes [60]. Another good flavor compound 5-methyl-2-phenyl-2-hexanl is a product formed via the Maillard reaction during roasting [54], which displays a deep bitter cocoa note with a sweet chocolate perception was found merely in the roasted rRC (0.56 μg/g) [56]. Surprisingly, the terpenes observed included β-myrcene, dℓ-limonene, 2-ethenylnaphthalene, alloocimene, and astonishingly, styrene and naphthalene (Table 9), inconsistent with the most of the literature that indicated the occurrence of geraniol, geranyl acetate, *α*-terpenyl formate, linalool and linalool derivatives (like cis-linalool oxide and trans-linalool oxide) [56]. Speculatively, the occurrence of styrene and naphthalenes was from the polluted soil. Worth mentioning, according to market information, although the organic products of chocolates still account for merely a small share of the total market, this part is steadily increasing [52].

In summary, the two coca bean cultivars in reality exhibited different compositions under the same treatment processes, as presented by fermentation, drying, and roasting in this study. These variations were significantly seen in the patterns relating with fatty acids, triacyglycerides, organic acids, amino acids, phenolics, flavonoids, and the volatiles. Contents of sugars and enzymic breakdown of polysaccharides form an important source of precursors [31,33,61]. Different cocoa bean genotypes or varieties influence both flavor quality and intensity in chocolate during manufacturing [1,18,33,61,62]. The differences are largely due to the wide differential in chemical composition of the derived beans, likely determining the quantities of flavor precursors and activity of enzymes, and thus contributions to flavor formation. More importantly, post-harvest processes (fermentation, drying, and roasting) have a strong influence on final flavors [1,31,33,61].

## 3. Materials and Methods

### 3.1. Source of Cocoa Pods and Processing

**Figure 1:**
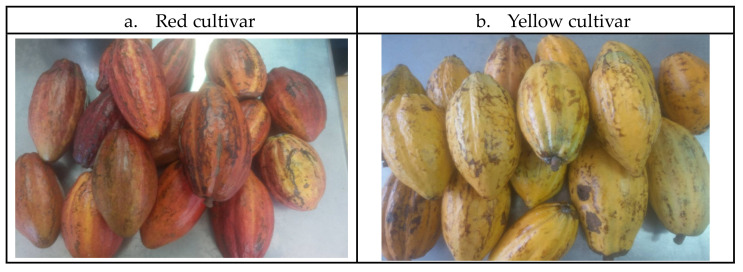
Cocoa pods from two different major cultivars grown in Taiwan.

Mature red (RC) and yellow (YC) cacao pods harvested from April 2018 to October 2019 were gifted by local farms in Taiwan (Figure 1).

**Figure 2:**
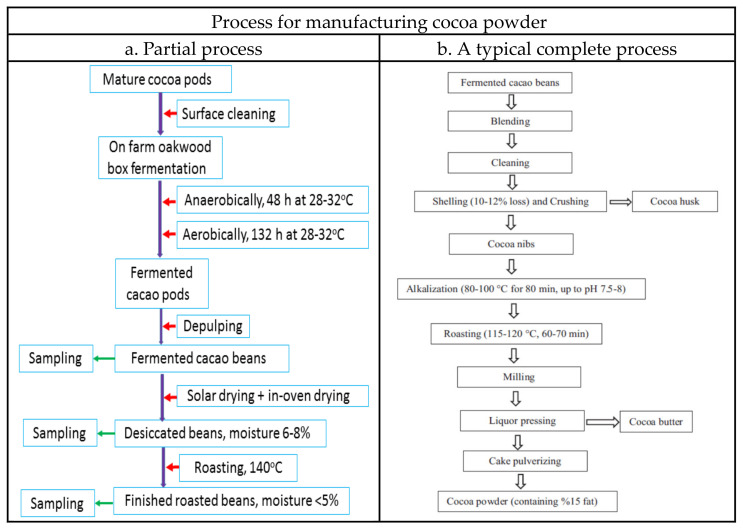
Flowchart for processing cocoa pods and cocoa beans. (**a**) Partial process for study in this experiment. (**b**) An example of the typical complete process [1]. Reprinted with permission from Ref. [1].

**Figure 3:**
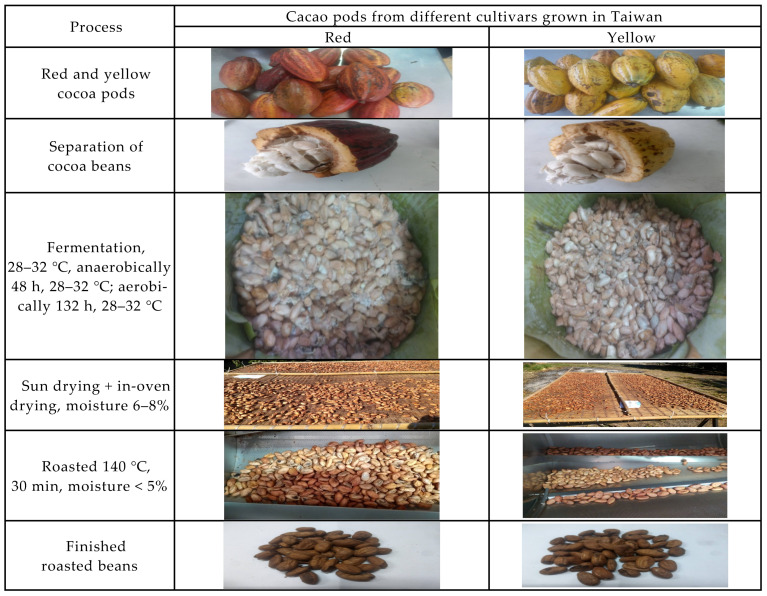
The three main processes (fermentation, drying, and roasting) for preparation of samples of roasted cocoa beans in this study.

Care was taken to only include fresh, undamaged pods. In this study, we mimic the traditional oakwood box fermentation with slight modification in order to reduce the problems coming from contamination and the uncertainty of fermentation conditions. These fresh cacao pods were cleaned, placed in tightly closed sterilized oakwood boxes (40 cm × 30 cm × 30 cm), anaerobically fermented for 48 h, and then aerobically for 132 h at 28–32 °C [34]. Depulping was immediately proceeded after opening of the fermented cocoa pods. The cocoa beans were solar dried (a traditional on-farm process) on the marquee and turned every 6 h during the first day, then with the assistance of in-over drying until reaching a moisture content between 6–8% [34], the dried cocoa beans were finally subjected to roasting at 140 °C until the moisture content was reduced to ≤5%. Thus, the fermented, the desiccated and the roasted beans were subjected to examination for the proximate composition, the patterns of fatty acid, triacylglycerides, organic acid, soluble sugars, umami tastes, antioxidant capability, and volatile compounds (see the flow chart Figure 2). Figure 3 shows the process-associated macro changes of fresh cocoa pods to roasted beans.

### 3.2. Proximate Analysis

AOAC was followed for analysis of the content of moisture, crude fat, crude protein, crude ash, and carbohydrate [63].

### 3.3. Determination of Fatty Acid Patterns

First, 0.1 g of crude fat was correctly measured and placed into a spiral test tube, 1 mL NaOH (1N) and 1 mL C-15 authentic sample solutions were added. The mixture was saponified at 85 °C for 15 min in the water bath, cooled, and 1 mL boron trifluoride (14%) was added and the derivation reaction was allowed to proceed on 100 °C water bath for 15 min [64]. After being cooled, 2 mL of n-hexane and 5 mL of saturated saline were added and left to stand until phase separated. The organic layer was separated and anhydrous sodium sulfate q.s. was added. The dehydrated organic phase was subjected to GC analysis. The analytical parameters of GC analysis with Agilent 6890 Series GC System were as follows: detector, FID type; analytical column, DB-1 (i.d. × ℓ = 0.25 mm × 60 m; thickness, 0.25 µm); carrier gas, and the N_2_-He-air mixture at a flow rate 1 mL/min. The temperature was programmed with an initial temperature 220 °C at an elevation rate 6 °C/min until 240 °C, then changed to 2 °C/min until 260 °C and maintained for 3 min.

### 3.4. Determination of Triacylglycerol Pattern

Method of AOCS was followed [65]. In brief, 0.2 g of sample was correctly measured and placed into a centrifuge tube, to which 1 mL hydrochloric acid and 5 mL dichloromethane were added and agitated for 5 min to mix well with the assistance of ultrasonication. The solution was centrifuged at 6000× *g* for 10 min. The bottom layer of dichloromethane extract was collected. Then, 10 μL of extract was measured and subjected to UPLC/ELSD analysis. Authentic triacylglycerols (TAGs) were used for calibration. The analytical column C-30 analytical column (Develosil C30-UG-5, 4.6 mm × 250 mm, 5 μm; Nomura Chemical Co., Anada-Cho Seto, Japan) was used and maintained at 30 °C. The temperature and the pressure of ELSD drift tube was maintained at 90 °C and 1.6 bar. The mobile phase consisted of different isopropanol (A) and acetonitrile (B), which were introduced at flow rate 1 mL/min in gradient elution manner: at time 0, A:B (in v%) = 40:60; at 25 min, A:B = 50:50; at 40 min, A:B = 60:40; at 50 min, A:B = 70:30; at 60 min, A:B = 40:60; and at 65 min, A:B = 40:60.

### 3.5. Analysis for the Umami Taste Compounds

The analysis for the umami taste compounds was performed, which included the organic acids, the soluble sugars, and the free amino acids.

#### 3.5.1. Determination of the Organic Acids

Samples 0.5~1.0 g were accurately measured, to which 50 mL of deionized water was added, magnetically stirred for 30 min, and centrifuged at 7155× *g* for 10 min. The supernatant was separated and filtered through a 0.22 μm micropore. The filtrate was subjected to HPC analysis against the authentic organic acids: oxalic, lactic, acetic, succinic, citric, malic, and tartaric acids. The separation column of HPLC was Luna^®^ 5µC18 (thickness = 5 μm, i.d. × ℓ = 4.6 mm × 250 mm). The detector used was HITACHI UV Detector L-2400. The mobile phase was 0.05 M potassium dihydrogen phosphate (pH 1.5). The flow rate was set at 1 mL/min for a duration of 20 min. The wave length for monitoring was 214 nm.

#### 3.5.2. Determination of the Soluble Sugars

The method was followed with a slight modification [66]. Briefly, 5 g of sample was accurately weighed to which 10 mL of 80% ethanol was added, after ultrasonicated at ambient temperature for 50 min, suction filtered through Whatman No. 4 filter paper. The residue was wetted five times with a total amount of 25 mL 80% ethanol. The combined filtrate was concentrated at 65 °C under reduced pressure until dried. The desiccated residue was redissolved in 20 mL deionized water, purified by filtering through 0.45 µm micropore, and subjected to HPLC analysis. Authentic solutions (maltose, lactose, glucose, fructose, mannitol, inositol, trehalose, xylose, and arabinose) of different concentrations were injected into HPLC for calibration. The analytical conditions of HPLC were as follows. The separation column used was Merck-LiChrospher^®^100, NH2 (5 μm, i.d. × ℓ = 4.6 mm × 250 mm), the detector, HITACHI-ELITE LaChrom. The mobile phase used was acetonitrile:water = 70:30 (*v/v*). The flow rate was set at 1 mL for a duration of 30 min.

#### 3.5.3. Determination of Free Amino Acids

The method of was slightly modified [67]. In brief, 0.1–1.0 g of sample was accurately weighed. To which 50 mL of 0.1 N HCl was added, agitated at ambient temperature for 45 min and filtered through Whatman No. 4 filter paper with the aid of suction. The filtrate was mixed with an equal amount of O-phthalaldehyde reagent in a test tube, agitated to facilitate the derivation reaction. The derived amino acids were subjected to HPLC analysis. The authentic amino acid solutions were used to calibrate the reference curve for each corresponding amino acid, from which the amount of each amino acid was obtained. The HPLC analysis was performed with the following conditions: The analytical column, LiChrospher 100 RP-C18 (i.d. × ℓ = 4.6 mm × 250 mm, thickness 5 um); the detector, HITACHI FL Detector L-2480. The mobile phase consisted of three solutions: solution A was 50 mM anhydrous sodium acetate (pH 5.7, containing 0.5% of tetrahydrofuran); solution B, 100% double filtered deionized water; solution C, 100% methanol. The flow rate was set at 1.0–1.2 mL/min maintained for 50 min. The detection wave length was 340 nm (excitation), and 450 nm (emission).

The gradient elution was set as (% ratio A:B:C; flow rate (fr), mL/min): at 0 min, 80:0:20, fr1.2; at 3 min, 76:0:24, fr1.2; at 10 min, 74:0:26; fr1.0; at 15 min, 70:0:30, fr1.0; at 20 min, 65:0:35, fr 1.1; at 22 min, 60:0:40, fr1.1; at 25 min, 50:0:50, fr 1.2; at 28 min, 50:0:50, fr 1.2; at 38 min, 33:0:67, fr1.0; at 40 min, 0: 33:67, fr1.2; at 43 min, 0:100:0, fr1.2; at 45 min, 0:100:0, fr1.2; at 46 min, 100:0:0, fr1.2; at 48 min, 80:0:20, fr1.2; at 50 min, 80:0:20, fr1.2.

### 3.6. Determination of Total Phenolics

Baba was followed with slight modification [68]. Briefly, to 1 mL of the sample solution, 1 mL of Folin–Ciocalteu reagent was added, agitated to mix well and left to stand for 3 min. Then, 0.1 mL 10% sodium carbonate was added and left to stand for 1 h. The absorbance was read against 735 nm. Gallic acid was used for establishing the calibration curve, from which the total phenolics were estimated and expressed in gallic acid equivalent (GAE) mg/mL.

### 3.7. Determination of Total Flavonoids

Baba was followed with slight modification [68]. In brief, to 1 mL of the extract, 1 mL of 2% methanolic AlCl_3_•6H_2_O was added, mixed well and left to stand for 10 min. The optical density was read against 430 nm. Quercetin was used to establish the calibration curve, from which the total flavonoid was calculated and expressed in quercetin equivalent (QE) in mg/mL.

### 3.8. Determination of Antioxidative Capability

#### 3.8.1. Preparation of Extracts

The cacao beans were grounded and pulverized, two 1 g of which were accurately weighed out and transferred, respectively, into the centrifugation tubes. To the two samples 10 mL of solvent ethanol (95%) and deionized water were, respectively, added, ultrasonicated for 2 h, and to each tube original fresh solvent 10 mL was added, ultrasonicated for 1 h, and filtered through 0.45 μm Whatman No. 4 filter paper with the aid of suction. The ethanolic and the aqueous extracts were, respectively, concentrated at 65 °C and 85 °C under vacuum until dry. The residues were, respectively, redissolved in the original fresh solvent to make up a final volume 10 mL, agitated well, decolorized with appropriate amount of active carbon, filtered through the 0.45 μm Whatman No. 4 filter paper with the aid of suction into the fresh centrifugation tubes, and subjected to the antioxidative assay.

#### 3.8.2. Determination of the Scavenging Capability for the DPPH Free Radicals

Lalhminghlui was followed with slight modification [69]. In brief, to different extracts (each 1 mL) 1 mL of DPPH (0.3 mM) methanolic solution was added, left to stand in the dark for 30 min. The absorbance was read against 517 nm. Standard Trolox solution was similarly treated to develop the calibration curve, which was used to calculate the scavenging capability for DPPH free radicals in terms of Trolox equivalent in mg/mL. Note, the slower the absorbance means the stronger the total antioxidative capability.

#### 3.8.3. Determination of the Scavenging Capability for the ABTS^+^ Free Radicals

Lalhminghlui was followed with slight modification [69]. Briefly, to prepare the ABTS free radical solution, 0.25 mL of 1000 μM ABTS was mixed with 1.5 mL deionized water, 0.25 mL 500 μM H_2_O_2_, and 0.25 mL peroxidase (44 U/mL) and left at ambient temperature for 30 min, avoiding direct sunlight to facilitate the production of bluish green ABTS^+^ solution. To 1.5 mL od ABTS^+^ solution different extract (each 0.05 mL) was added and left to stand for 5 min. The absorbance was read against 734 nm. Standard Trolox solution was similar treated to develop the calibration curve, which was used to calculate the scavenging capability for ABTS^+^ free radicals in terms of Trolox equivalent in mg/mL. Note, the slower the absorbance means the stronger the total antioxidative capability.

### 3.9. Determination of the Volatile Compounds

The so-called solid phase microextraction method (SPME) was used [70]. Briefly, 5 g of samples were accurately measured and transferred into a 20 mL-sample vial and sealed with aluminum foil through which the microextractor needle was inserted with the height of the adsorption fiber appropriately adjusted. The adsorption of the volatiles was proceeded at least for 30 min and subjected to GC/MS analysis using Agilent Model 6890 GC (SantaClara, CA, USA). The analytical conditions were as follows: The separation column: Agilent DB-wax (i.d. × ℓ = 0.25 mm × 60 m, thickness = 0.25 µm). The temperatures at the inlet and detector were 250 and 280 °C, respectively. The ionization voltage was 70 V and the ion source temperature was 230 °C. The carrier gas He was set at a flow rate of 1 mL/min. The split ratio was splitless for SPME unlike 80:1 in general cases. The oven temperature was programmed as: The initial temperature was 40 °C at 0 min and the elevation rate was set at 3 °C/min until 150 °C, remained for 0 min. Then, the elevation rate was changed to 5 °C/min until 220 °C and maintained for 30 min. For introduction of the samples, the microextractor was inserted at the injection port for 15 min to facilitate the desorption.

### 3.10. Determination of the Retention Index (RI)

The GC retention index of volatiles was determined by referring to the RI indices of mixed C5~C25 normal alkane authentic solutions obtained by similar treatments. The non-isothermal Kovats retention indices (from temperature-programming) was expressed as:

RI_x_ = 100n + 100(t_x_ − t_n_)/(t_n+1_ − t_n_) (1)

where t_n_ and t_n+1_ are retention times of the reference n-alkane hydrocarbons eluting immediately before and after chemical compound “X”; t_x_ is the retention time of compound “X”.

### 3.11. Statistical Analysis

Data were collected from triplet experiments and expressed as mean ± SD. The computer statistical software SPSS18 (SPSS, Chicago, IL, USA) was adopted. The analysis of variance (ANOVA) was used to test the variation within the same group. Duncan’s multiple range test was used to compare the statistical significance of difference among groups. The independent student *t*-test was used to statistically compare the significance of difference between two groups. A difference with *p* < 0.05 was considered to be statistically significant.

## 4. Conclusions

The data presented here has shown that the red and the yellow cultivars are totally different varieties. Different cocoa cultivars contain different biochemical compounds (storage proteins, fatty acids, triacylglycerols, carbohydrates, amino acids, and polyphenols), which may give off different flavors and umami compounds even treated with similar processes including fermentation, drying, and roasting. More importantly, fermentation is the crucial step that determines many biochemical metabolic pathways, converting the biochemical constituents into a diversity of fatty acids, organic acids, and amino acids. As most of the fermentations and drying of coca pods are traditionally carried out in an on-farm in-box fermentation and on-marquee solar drying manner, obviously the conditions would vary a great deal from batch to batch, as well as from farm to farm, regarding the fermentation temperature, oxygen tension, and the microbial spectra. Our findings have demonstrated that it is possible to maintain or increase the biological activity of cocoa beans and their derived products (cocoa powder and chocolate) by choosing appropriate processing conditions and cocoa genotype, i.e., in order to ensure a characteristically favorite cocoa quality, careful control of the processes of fermentation, drying, and roasting with respect to the cultivar’s characteristics will lead to improved products in the end.

## Figures and Tables

**Table 1:** Variation of proximate composition in differently treated polymorphic cacao beans *.

Group	Content (%)				
	Moisture	Crude Ash	Crude Protein	Crude Fat	Carbohydrate
fRC	9.31 ± 0.16 ^b^	3.24 ± 0.00 ^d^	11.51 ± 0.60 ^b^	41.70 ± 0.50 ^d^	34.24
dRC	5.60 ± 0.07 ^c^	3.51 ± 0.01 ^b^	13.20 ± 0.01 ^a^	43.86 ± 0.19 ^bc^	33.83
rRC	1.00 ± 0.03 ^f^	3.69 ± 0.01 ^a^	13.43 ± 0.01 ^a^	50.03 ± 0.64 ^a^	31.85
fYC	9.59 ± 0.02 ^a^	3.04 ± 0.00 ^e^	13.91 ± 0.32 ^a^	40.52 ± 0.44 ^e^	32.94
dYC	5.11 ± 0.03 ^d^	3.38 ± 0.06 ^c^	13.66 ± 0.06 ^a^	43.13 ± 0.32 ^c^	34.72
rYC	1.26 ± 0.18 ^e^	3.69 ± 0.07 ^a^	13.33 ± 0.35 ^a^	44.49 ± 0.71 ^b^	37.23

* Each value is expressed as mean ± SD (*n* = 3). Means with different superscripts in lower case within the same column are significantly different from each other (*p* < 0.05). RC: red cocoa. YC: yellow cocoa. The heading letters f: fermented. d: desiccated. r: roasted.

**Table 2:** Variation of fatty acid pattern in differently treated polymorphic cacao beans *.

Group	Content (%)			
	Palmitic Acid (16:0)	Stearic Acid (18:0)	Oleic Acid (18:1)	Arachidic Acid (20:0)
fRC	0.75 ± 0.01 ^c^	1.06 ± 0.04 ^c^	0.60 ± 0.03 ^c^	0.12 ± 0.03 ^ab^
dRC	0.83 ± 0.03 ^b^	1.16 ± 0.03 ^b^	0.76 ± 0.04 ^b^	0.13 ± 0.03 ^b^
rRC	0.75 ± 0.01 ^c^	0.93 ± 0.01 ^d^	0.72 ± 0.03 ^b^	n.d.
fYC	0.57 ± 0.04 ^d^	0.70 ± 0.03 ^e^	0.57 ± 0.01 ^c^	0.08 ± 0.03 ^ab^
dYC	0.96 ± 0.03 ^a^	1.28 ± 0.03 ^a^	0.93 ± 0.04 ^a^	0.15 ± 0.04 ^a^
rYC	0.25 ± 0.04 ^e^	0.34 ± 0.06 ^f^	0.24 ± 0.03 ^d^	0.05 ± 0.01 ^b^

* Each value is expressed as mean ± SD (*n* = 3). Means with different superscripts in lower case within the same column are significantly different from each other (*p* < 0.05). RC: red cocoa. YC: yellow cocoa. The heading letters f: fermented. d: desiccated. r: roasted.

**Table 3:** Variation of triacylglyceride pattern in differently treated polymorphic cacao beans *.

Group	Content (%)					
	POP	POS	OOO	SOO	SOA	SOS
fRC	16.91 ± 0.04 ^c^	53.69 ± 0.02 ^b^	0.64 ± 0.01 ^e^	1.30 ± 0.10 ^d^	0.29 ± 0.04 ^ab^	26.99 ± 0.04 ^d^
dRC	16.34 ± 0.02 ^d^	53.23 ± 0.08 ^c^	0.77 ± 0.03 ^d^	1.57 ± 0.06 ^c^	0.31 ± 0.07 ^a^	27.50 ± 0.07 ^b^
rRC	19.27 ± 0.02 ^a^	55.43 ± 0.13 ^a^	0.83 ± 0.03 ^c^	1.18 ± 0.06 ^e^	0.14 ± 0.06 ^d^	22.81 ± 0.05 ^f^
fYC	16.96 ± 0.02 ^b^	53.08 ± 0.07 ^d^	0.87 ± 0.02 ^b^	1.71 ± 0.03 ^b^	0.27 ± 0.05 ^bc^	26.80 ± 0.12 ^e^
dYC	15.59 ± 0.03 ^f^	52.95 ± 0.08 ^f^	0.88 ± 0.04 ^b^	1.70 ± 0.02 ^b^	0.24 ± 0.09 ^c^	28.27 ± 0.09 ^a^
rYC	16.18 ± 0.02 ^e^	53.01 ± 0.09 ^e^	1.09 ± 0.05 ^a^	1.94 ± 0.04 ^a^	0.26 ± 0.09 ^bc^	27.21 ± 0.06 ^c^

* Each value is expressed as mean ± SD (*n* = 3). Means with different superscripts in lower case within the same column are significantly different from each other (*p* < 0.05). RC: red cocoa. YC: yellow cocoa. The heading letters f: fermented. d: desiccated. r: roasted.

**Table 4:** Variation of organic acid pattern in differently treated polymorphic cacao beans *.

Group	Content (mg/100 g)				
	Citric Acid	Tartaric Acid	Succinic Acid	Acetic Acid	Malic Acid
fRC	n.d.	50.59 ± 0.25 ^c^	n.d.	0.30 ± 0.04 ^f^	1.73 ± 0.06 ^f^
dRC	177.63 ± 0.18 ^b^	25.45 ± 0.03 ^c^	18.32 ± 0.05 ^c^	86.43 ± 0.20 ^b^	74.32 ± 0.15 ^d^
rRC	39.58 ± 0.12 ^c^	7.12 ± 0.05 ^d^	19.61 ± 0.10 ^b^	33.99 ± 0.16 ^e^	89.59 ± 0.08 ^b^
fYC	37.28 ± 0.08 ^d^	80.31 ± 0.14 ^b^	7.43 ± 0.06 ^d^	57.26 ± 0.07 ^d^	79.26 ± 0.14 ^c^
dYC	34.78 ± 0.09 ^e^	80.62 ± 0.12 ^b^	7.39 ± 0.27 ^d^	79.04 ± 0.08 ^c^	15.35 ± 0.12 ^e^
rYC	335.56 ± 0.19 ^a^	99.29 ± 0.08 ^a^	36.42 ± 0.10 ^a^	720.48 ± 0.08 ^a^	129.32 ± 0.05 ^a^

* Each value is expressed as mean ± SD (*n* = 3). Means with different superscripts in lower case within the same column are significantly different from each other (*p* < 0.05). RC: red cocoa. YC: yellow cocoa. The heading letters f: fermented. d: desiccated. r: roasted.

**Table 5:** Variation of soluble sugar pattern in differently treated polymorphic cacao beans *.

Group	Sugar, (g/100 g)	
	Fructose	Glucose
fRC	1.480	0.735
dRC	n.d.	0.404
rRC	n.d.	n.d.
fYC	1.483	n.d.
dYC	n.d.	n.d.
rYC	n.d.	n.d.

* Each value is expressed as mean ± SD (*n* = 3). RC: red cocoa. YC: yellow cocoa. The heading letters f: fermented. d: desiccated. r: roasted.

**Table 6:** Variation of amino acid pattern in differently treated polymorphic cacao beans *.

Amino Acid (mg/100 g)	Group					
	fRC	dRC	rRC	fYC	dYC	rYC
Aspartic acid	35.25 ± 0.03 ^a^	33.86 ± 0.02 ^b^	17.76 ± 0.06 ^f^	27.38 ± 0.07 ^d^	29.34 ± 0.04 ^c^	21.88 ± 0.09 ^e^
Glutamic acid	61.76 ± 0.02 ^d^	65.60 ± 0.06 ^c^	10.94 ± 0.05 ^f^	79.39 ± 0.07 ^a^	77.58 ± 0.08 ^b^	15.90 ± 0.04 ^e^
Asparagine	65.07 ± 0.02 ^d^	80.78 ± 0.06 ^a^	17.71 ± 0.05 ^f^	70.49 ± 0.07 ^c^	75.20 ± 0.08 ^b^	25.67 ± 0.04 ^e^
Serine	41.42 ± 0.04 ^a^	40.52 ± 0.05 ^b^	16.31 ± 0.03 ^f^	33.82 ± 0.05 ^c^	28.17 ± 0.04 ^d^	18.41 ± 0.05 ^e^
Glutamine	24.90 ± 0.04 ^a^	22.60 ± 0.05 ^b^	n.d.	17.31 ± 0.10 ^d^	17.95 ± 0.04 ^c^	n.d.
Histidine	7.96 ± 0.08 ^c^	8.26 ± 0.03 ^a^	n.d.	4.90 ± 0.05 ^d^	n.d.	8.13 ± 0.05 ^b^
Glycine	29.78 ± 0.05 ^a^	28.09 ± 0.02 ^b^	20.29 ± 0.02 ^d^	22.78 ± 0.03 ^c^	19.07 ± 0.03 ^e^	14.41 ± 0.02 ^f^
Threonine	31.31 ± 0.03 ^a^	27.16 ± 0.04 ^b^	11.32 ± 0.04 ^e^	22.91 ± 0.04 ^c^	16.26 ± 0.03 ^d^	9.43 ± 0.03 ^f^
Arginine	67.93 ± 0.04 ^a^	64.62 ± 0.04 ^b^	18.16 ± 0.13 ^e^	56.90 ± 0.04 ^c^	42.75 ± 0.03 ^d^	11.30 ± 0.03 ^f^
Alanine	61.17 ± 0.05 ^a^	60.66 ± 0.02 ^b^	22.09 ± 0.06 ^f^	60.42 ± 0.02 ^c^	49.41 ± 0.04 ^d^	23.56 ± 0.02 ^e^
Tryosine	50.28 ± 0.04 ^a^	47.59 ± 0.04 ^b^	16.77 ± 0.05 ^e^	38.69 ± 0.04 ^c^	31.47 ± 0.03 ^d^	15.85 ± 0.02 ^f^
Cystine	14.88 ± 0.04 ^a^	7.18 ± 0.02 ^b^	n.d.	n.d.	n.d.	n.d.
Valine	51.90 ± 0.04 ^b^	52.92 ± 0.02 ^a^	18.22 ± 0.04 ^f^	42.79 ± 0.02 ^c^	37.37 ± 0.04 ^d^	19.40 ± 0.03 ^e^
Methionine	23.62 ± 0.02 ^a^	19.29 ± 0.03 ^b^	14.18 ± 0.06 ^c^	11.09 ± 0.02 ^d^	n.d.	n.d.
Tryptophan	114.13 ± 0.04 ^b^	133.88 ± 0.01 ^a^	19..28 ± 0.04 ^e^	53.61 ± 0.03 ^d^	56.16 ± 0.03 ^c^	16.78 ± 0.03 ^f^
Phenylalanine	91.93 ± 0.02 ^b^	93.18 ± 0.03 ^a^	17.21 ± 0.05 ^e^	73.41 ± 0.01 ^c^	55.89 ± 0.04 ^d^	15.42 ± 0.03 ^f^
Isoleucine	44.39 ± 0.02 ^b^	44.90 ± 0.03 ^a^	9.50 ± 0.04 ^f^	33.88 ± 0.03 ^c^	29.50 ± 0.02 ^d^	14.21 ± 0.05 ^e^
Leucine	82.39 ± 0.02 ^a^	81.81 ± 0.02 ^b^	2.47 ± 0.05 ^e^	66.22 ± 0.03 ^c^	46.31 ± 0.02 ^d^	n.d.
Lysine	109.11 ± 0.05 ^a^	77.82 ± 0.04 ^b^	19.02 ± 0.02 ^e^	70.88 ± 0.03 ^c^	55.68 ± 0.02 ^d^	17.46 ± 0.04 ^f^
Proline	95.07 ± 0.02 ^a^	n.d.	n.d.	n.d.	n.d.	n.d.
Total	1104.25 ± 0.71 ^a^	990.72 ± 0.63 ^b^	231.95 ± 0.79 ^f^	786.87 ± 0.75 ^c^	668.11 ± 0.61 ^d^	247.81 ± 0.57 ^e^

* Each value is expressed as mean ± SD (*n* = 3). Means with different superscripts in lower case within the same row are significantly different from each other (*p* < 0.05). RC: red cocoa. YC: yellow cocoa. The heading letters f: fermented. d: desiccated. r: roasted.

**Table 7:** Variation of total phenolic and flavonoid pattern in differently treated polymorphic cacao beans *.

Group	TP, (Gallic Acid Eq. μg/g)		TF, (Quercetin Eq. μg/g)	
	Water	EtOH	Water	EtOH
fRC	^B^8.94 ± 0.09 ^a^	^A^16.35 ± 0.04 ^c^	^B^2.60 ± 0.02 ^a^	^A^14.46 ± 0.06 ^a^
dRC	^B^4.74 ± 0.02 ^c^	^A^12.65 ± 0.08 ^d^	^B^2.35 ± 0.04 ^b^	^A^9.14 ± 0.06 ^b^
rRC	^B^4.66 ± 0.11 ^c^	^A^8.65 ± 0.06 ^f^	^B^ 2.04 ± 0.08 ^c^	^A^9.00 ± 0.02 ^c^
fYC	^B^6.52 ± 0.06 ^b^	^A^20.15 ± 0.11 ^a^	^B^2.63 ± 0.02 ^a^	^A^8.84 ± 0.08 ^d^
dYC	^B^3.85 ± 0.05 ^d^	^A^18.61 ± 0.23 ^b^	^B^1.86 ± 0.02 ^d^	^A^8.63 ± 0.02 ^e^
rYC	^B^2.54 ± 0.11 ^e^	^A^10.85 ± 0.07 ^e^	^B^1.85 ± 0.02 ^d^	^A^5.90 ± 0.04 ^f^

* Each value is expressed as mean ± SD (*n* = 3). Means with different superscripts in lower case within the same column are significantly different from each other (*p* < 0.05). The means with different superscripts in upper case within the same row are significantly different from each other (*p* < 0.05). RC: red cocoa. YC: yellow cocoa. The heading letters f: fermented. d: desiccated. r: roasted.

**Table 8:** Variation of the DPPH and ABTS free radical scavenging capabilities of differently treated polymorphic cacao beans *.

Group	DPPH (Trolox mg/100 g)		ABTS (Trolox mg/100 g)	
	Water	EtOH	Water	EtOH
fRC	^B^1.95 ± 0.03 ^cd^	^A^2.27 ± 0.18 ^a^	^B^35.22 ± 0.04 ^a^	^A^37.89 ± 0.06 ^a^
dRC	^B^1.92 ± 0.08 ^d^	^A^2.16 ± 0.06 ^c^	^B^35.21 ± 0.12 ^a^	^A^36.59 ± 0.04 ^b^
rRC	^B^1.82 ± 0.03 ^e^	^A^2.03 ± 0.12 ^f^	^B^34.01 ± 0.04 ^e^	^A^36.19 ± 0.04 ^c^
fYC	^B^2.04 ± 0.04 ^a^	^A^2.22 ± 0.08 ^b^	^B^34.93 ± 0.04 ^b^	^A^36.16 ± 0.08 ^c^
dYC	^B^2.00 ± 0.09 ^b^	^A^2.08 ± 0.05 ^d^	^B^34.70 ± 0.04 ^c^	^A^35.44 ± 0.04 ^d^
rYC	^B^1.95 ± 0.06 ^c^	^A^2.06 ± 0.05 ^e^	^B^34.50 ± 0.08 ^d^	^A^35.39 ± 0.04 ^d^

* Each value is expressed as mean ± SD (*n* = 3). Means with superscripts in different lower case within the same column are significantly different from each other (*p* < 0.05). The means with superscripts in different upper case within the same row are significantly different from each other (*p* < 0.05). RC: red cocoa. YC: yellow cocoa. The heading letters f: fermented. d: desiccated. r: roasted.

**Table 9:** Volatile patterns identified in differently treated polymorphic cacao beans *.

Compounds	RI	Formula	M.W.	CAS NO.
Alcohols				
Isobutanol	1014	C_4_H_10_O	74	78-83-1
2-Pentanol	1045	C_5_H_12_O	88	6032-29-7
Isoamyl alcohol	1124	C_5_H_12_O	88	123-51-3
3-Methyl-3-Buten-1-ol	1158	C_5_H_10_O	86	763-32-6
2-Heptanol	1239	C_10_H_20_O_2_	172	25415-62-7
3-Ethyl-2-pentanol	1254	C_7_H_16_O	116	609-27-8
3-Ethoxy-1-propanol	1275	C_5_H_12_O_2_	104	111-35-3
2-Butoxyethanol	1301	C_6_H_14_O_2_	118	111-76-2
2-Octanol	1333	C_8_H_18_O	130	123-96-6
2-Ethylhexanol	1394	C_8_H_18_O	130	104-76-7
[S-(R*,R*)]-2,3-Butanediol	1415	C_7_H_6_O	106	100-52-7
2-Nonanol	1429	C_9_H_20_O	144	628-99-9
Linalool	1448	C_10_H_18_O	154	78-70-6
2-Octanol	1458	C_8_H_18_O	130	123-96-6
Fenchyl alcohol	1482	C_10_H_18_O	154	1632-73-1
2-(2-Ethoxyethoxy)ethanol	1494	C_6_H_14_O_3_	134	111-90-0
Furfuryl alcohol	1516	C_5_H_6_O_2_	98	98-00-0
Methionol	1579	C_4_H_10_OS	104	505-10-2
α-Terpineol	1587	C_10_H_18_O	154	98-55-5
Epoxylinalol	1641	C_10_H_18_O_2_	170	14049-11-7
Butyl carbitol	1668	C_8_H_18_O_3_	162	112-34-5
1-Phenethyl alcohol	1672	C_8_H_10_O	122	1517-69-7
Benzyl alcohol	1723	C_7_H_8_O	108	100-51-6
2-Phenylethyl Alcohol	1759	C_8_H10O	122	98-85-1
1-Phenoxy-2-propanol	1876	C_9_H_12_O_2_	152	770-35-4
**Aldehydes**				
2-Methyl propanal	842	C_4_H_8_O	72	78-84-2
3-Methyl butanal	880	C_5_H_10_O	86	590-86-3
trans-5-Methyl-2-isopropyl-2-hexen-1-al	1288	C_10_H_18_O	154	0000-00-0
Furfural	1341	C_5_H_4_O_2_	96	98-01-1
Benzaldehydehyde	1405	C_11_H_22_O_2_	186	2461-15-6
Benzaldehyde	1405	C_7_H_6_O	106	100-52-7
Benzeneacetaldehyde	1510	C_8_H_8_O	120	122-78-1
2-Phenyl-2-butenal	1785	C_10_H_10_O	146	4411-89-6
5-Methyl-2-phenyl-2-hexenal	1925	C_13_H_16_O	188	21834-92-4
**Ketones**				
1-Methoxy-2-propanone	885	C_4_H_8_O_2_	88	5878-19-3
2-Pentanone	913	C_5_H_10_O	86	107-87-9
2-Heptanone	1112	C_7_H_14_O	114	110-43-0
2-Methyltetrahydro 3-furanone	1168	C_5_H_8_O_2_	100	3188-00-9
Acetoin	1182	C_12_H_14_O_2_	190	63678-00-2
3-Hepten-2-one	1219	C_8_H_16_O_2_	144	25368-54-1
2-Nonanone	1311	C_9_H_18_O	142	821-55-6
Acetoxyacetone	1345	C_5_H_8_O_3_	116	592-20-1
Butyrolactone	1487	C_4_H_6_O_2_	86	96-48-0
Acetophenone	1524	C_8_H_8_O	120	98-86-2
Pantolactone	1849	C_6_H_10_O_3_	130	599-04-2
5-Acetyldihydro-2(3H)-furanone	1875	C_6_H_8_O_3_	128	29393-32-6
3,5-Dihydroxy-6- methyl-2,3-dihydro-4H-pyran-4-one	2052	C_6_H_8_O_4_	144	28564-83-2
**Acids**				
Acetic acid	1324	C_2_H_4_O_2_	60	64-19-7
Isobutyric acid	1441	C_4_H_8_O_2_	88	79-31-2
Isovaleric acid	1487	C_5_H_10_O_2_	102	503-74-2
Valeric acid	1534	C_5_H_10_O_2_	102	109-52-4
**Esters**				
Ethyl acetate	866	C_4_H_8_O_2_	88	141-78-6
Isobutyl acetate	955	C_7_H_14_O_2_	130	1561-11-1
Isoamyl acetate	1057	C_7_H_14_O_2_	130	123-92-2
Dimethyl sulfuroate	1098	C_2_H_6_O_3_S	110	616-42-2
Ethyl hexanoate	1166	C_8_H_16_O_2_	144	123-66-0
1-Methylhexyl acetate	1196	C_9_H_18_O_2_	158	5921-82-4
Isoamyl butyrate	1201	C_9_H_18_O_2_	158	60415-61-4
n-Amyl isovalerate	1234	C_7_H_12_O	112	1119-44-4
Isobutyl hexanoate	1285	C_10_H_20_O_2_	172	105-79-3
Ethyl octanoate	1361	C_10_H_20_O_2_	172	106-32-1
Isopentyl hexanoate	1387	C_11_H_22_O_2_	186	2198-61-0
Ethyl benzoate	1549	C_9_H_10_O_2_	150	093-89-0
Methyl phenylacetate	1627	C_9_H_10_O_2_	150	101-41-7
β-Phenethylformate	1652	C_9_H_10_O_2_	150	104-62-1
4-Ethylphenyl acetate	1659	C_10_H_12_O_2_	164	3245-23-6
2-Phenylethylacetate	1686	C_10_H_12_O_2_	164	103-45-7
Isobutyl benzoate	1725	C_11_H_14_O_2_	178	120-50-3
**Terpenes**				
β-Myrcene	1105	C_10_H_16_	136	123-35-3
dℓ-Limonene	1145	C_10_H_16_	136	138-86-3
Styrene	1174	C_8_H_8_	104	100-42-5
Alloocimene	1298	C_10_H_16_	136	0000-00-0
cis-Linalool oxide	1355	C_10_H_18_O_2_	170	5989-33-3
trans-Linalool oxide	1380	C_10_H_18_O_2_	170	34995-77-2
Naphthalene	1614	C_10_H_8_	128	91-20-3
2-Ethenyl-naphthalene	1845	C_12_H_10_	154	827-54-3
**Pyrazines**				
Methylpyrazine	1176	C_5_H_6_N_2_	94	109-08-0
2,5-Dimethylpyrazine	1236	C_6_H_8_N_2_	108	123-32-0
2,6-Dimethylpyrazine	1241	C_6_H_8_N_2_	108	108-50-9
Ethylpyrazine	1244	C_6_H_8_N_2_	108	13925-00-3
2,3-Dimethylpyrazine	1257	C_6_H_8_N_2_	108	5910-89-4
2-Ethyl-6-methyl-pyrazine	1295	C_7_H_10_N_2_	122	13925-03-6
2-Methyl-5-ethylpyrazine	1301	C_7_H_10_N_2_	122	13360-64-0
Trimethylpyrazine	1315	C_7_H_10_N_2_	122	14667-55-1
2,6-Diethyl-pyrazine	1347	C_8_H_12_N_2_	136	13067-27-1
2-Ethyl-3,5-dimethyl pyrazine	1359	C_8_H_12_N_2_	136	13925-07-0
2-Ethyl-3,6-dimethylpyrazine	1359	C_8_H_12_N_2_	136	013360-65-1
2,5-Diethylpyrazine	1370	C_8_H_12_N_2_	136	13238-84-1
2,5-Diethylpyrazine	1373	C_8_H_12_N_2_	136	13067-27-1
5-Ethyl-2,3-dimethylpyrazine	1374	C_8_H_12_N_2_	136	15707-34-3
Tetramethylpyrazine	1386	C_8_H_12_N_2_	170	1124-11-4
3,5-Diethyl-2-methyl-pyrazine	1407	C_9_H_14_N_2_	150	18138-05-1
2,3,5-Ttrimethyl-6-ethylpyrazine	1426	C_9_H_14_N_2_	150	17398-16-2
**Others**				
2,3-Dihydrofuran	1183	C_4_H_6_O	70	1191-99-7
2,5Diethyltetrahydrofuran	1285	C_8_H_16_O	128	41239-48-9
2-Acetylfuran	1384	C_6_H_6_O_2_	110	1192-62-7
Propyl nitrite	1453	C_3_H_7_NO_2_	89	543-67-9
2-Acetylpyrrole	1800	C_6_H_7_NO	109	1072-83-9
Benzothiazole	1801	C_7_H_5_NS	135	95-16-9
Phenol	1827	C_6_H_6_O	94	108-95-2

* Each value is expressed as mean ± SD (*n* = 3).

**Table 10:** Variation of volatile patterns in differently treated polymorphic cacao beans *.

Compounds	Composition (μg/g)					
	fRC	dRC	rRC	fYC	dYC	rYC
Alcohols						
Isobutanol	43.80	8.35	3.92	10.71	28.53	28.30
2-Pentanol	24.22	4.30	3.53	1.77	4.36	5.33
Isoamyl alcohol	214.73	36.05	16.22	59.80	135.94	130.92
3-Methyl-3-Buten-1-ol	-	-	-	-	-	1.09
2-Heptanol	109.52	13.28	25.31	1.57	2.60	-
3-Ethyl-2-pentanol	-	-	1.73	-	-	-
3-Ethoxy-1-propanol	-	-	-	0.18	0.75	0.99
2-Butoxyethanol	-	-	-	0.34	-	-
2-Octanol	9.24	1.37	2.16	0.31	0.58	1.29
2-Ethylhexanol	2.17	0.24	0.49	0.42	0.44	0.51
[S-(R*,R*)]-2,3- Butanediol	15.80	3.75	3.79	2.07	5.89	4.56
2-Nonanol	46.39	7.28	15.17	0.15	0.72	0.43
Linalool	20.90	3.28	14.31	2.25	7.00	10.10
2-Octanol	-	-	1.56	-	-	-
Fenchyl alcohol	1.56	0.22	0.75	0.22	0.27	0.50
2-(2-Ethoxyethoxy) ethanol	-	-	-	0.27	0.21	0.38
Furfuryl alcohol	-	-	6.21	-	-	5.12
Methionol	-	-	-	0.19	-	2.09
α-Terpineol	6.91	1.30	3.39	1.17	2.04	3.11
Epoxylinalol	-	0.17	0.19	-	-	0.33
Butyl carbitol	-	-	-	0.20	-	-
1-Phenethyl alcohol	2.01	0.30	0.44	0.16	0.29	0.41
Benzyl alcohol	1.91	0.31	0.28	0.30	0.39	0.55
2-Phenylethyl Alcohol	344.95	56.81	53.89	54.47	124.94	171.54
1-Phenoxy-2- propanol	-	-	-	1.18	0.25	0.38
**subtotal**	**844.11**	**137.01**	**153.34**	**137.73**	**315.2**	**367.93**
**Aldehydes**						
2-Methylpropanal	9.56	1.12	12.66	2.52	4.57	18.50
3-Methylbutanal	103.97	24.29	89.15	-	-	-
trans-5-Methyl-2-isopropyl-2-hexen-1-al	1.29	-	1.83	-	-	-
Furfural	5.58	0.94	13.92	0.47	0.74	5.89
Benzaldehydehyde	23.96	4.04	3.71	-	-	-
Benzaldehyde	-	-	-	3.11	7.81	10.47
Benzeneacetaldehyde	50.39	6.52	5.00	3.58	5.08	7.25
2-Phenyl-2-butenal	-	-	1.50	0.10	0.33	2.15
5-Methyl-2-phenyl-2-hexenal	-	-	0.56	-	-	-
**subtotal**	**194.75**	**36.91**	**128.33**	**9.78**	**18.53**	**44.26**
**Ketones**						
1-Methoxy-2- propanone	-	-	-	37.57	99.59	194.84
2-Pentanone	42.00	11.12	5.42	7.23	21.10	14.16
2-Heptanone	79.36	11.23	9.92	1.53	2.62	1.33
Acetoin	23.73	3.89	-	2.24	8.15	5.87
2-Methyltetrahydro 3-furanone	-	-	1.19	-	-	-
3-Hepten-2-one	2.95	0.46	1.23	-	-	-
2-Nonanone	40.64	8.30	22.00	0.53	1.29	0.95
Acetoxyacetone	-	-	1.10	-	-	-
Butyrolactone	-	-	-	0.28	0.68	3.39
Acetophenone	7.03	1.15	0.98	0.57	1.09	1.47
Pantolactone	1.84	0.21	1.11	0.16	0.23	0.29
5-Acetyldihydro-2(3H)-furanone	-	-	0.56	-	-	-
3,5-Dihydroxy-6- methyl-2,3-dihydro-4H-pyran-4-one	-	-	0.49	-	-	-
**subtotal**	**197.55**	**36.36**	**44.00**	**50.11**	**134.75**	**222.30**
**Acids**						
Acetic acid	29.68	4.56	12.99	0.89	3.75	7.92
Isobutyric acid	56.45	9.75	15.17	-	-	-
Isovaleric acid	3.55	0.50	3.96	-	-	-
Valeric acid	-	28.88	41.98	0.60	1.91	3.70
**subtotal**	**89.68**	**43.69**	**74.1**	**1.49**	**5.66**	**11.62**
**Esters**						
Ethyl acetate	-	-	-	5.48	16.24	51.18
Isobutyl acetate	3.94	1.74	-	3.66	8.80	11.25
Isoamyl acetate	101.26	15.69	9.27	24.65	47.76	65.80
Dimethyl sulfuroate	4.87	0.66	1.53	0.47	0.65	1.73
Ethy hexanoate	8.15	1.33	1.22	1.38	3.76	6.65
1-Methylhexyl acetate	9.14	1.15	3.53	-	0.41	-
Isoamyl butyrate	-	0.21	-	0.42	0.75	1.68
n-Amyl isovalerate	3.80	0.53	0.39	-	0.29	-
Isobutyl hexanoate	-	-	-	0.36	0.74	0.95
Ethyl octanoate	-	0.86	1.31	1.47	2.60	5.34
Isopentyl hexanoate	-	-	-	0.29	0.79	2.29
Ethyl benzoate	-	-	1.56	0.43	1.03	1.51
Methyl phenylacetate	-	-	0.35	-	-	-
β-Phenethyl formate	-	-	-	--	-	0.44
4-Ethylphenyl acetate	1.24	0.24	0.62	0.27	0.49	0.84
2-Phenylethyl acetate	22.11	3.57	5.37	3.69	8.30	14.62
Isobutyl benzoate	1.25	0.29	045	0.19	0.22	0.36
**subtotal**	**155.76**	**26.27**	**70.15**	**42.76**	**92.83**	**164.64**
**Terpenes**						
β-Myrcene	2.84	0.36	-	0.32	0.96	3.85
dℓ-Limonene	-	-	-	0.50	-	-
Styrene	8.84	0.93	-	1.33	2.56	-
Alloocimene	-	-	-	-	0.26	-
cis-Linalool oxide	2.33	0.37	0.81	0.27	0.55	0.89
trans-Linalool oxide	5.45	0.78	0.97	0.48	1.22	1.76
Naphthalene	-	-	-	0.17	0.19	-
1-Ethenyl- naphthalene	-	-	-	0.09	-	0.78
**subtotal**	**19.46**	**2.44**	**1.78**	**3.16**	**8.90**	**7.28**
**Pyrazines**						
Methyl-pyrazine	-	-	9.75	-	-	17.07
2,5-Dimethyl- pyrazine	-	-	-	-	-	14.64
2,6-Dimethyl- pyrazine	-	-	-	-	-	6.40
Ethylpyrazine	-	-	2.73	-	-	3.95
2,3-Dimethylpyrazine	-	-	0.42	-	-	2.77
2-Ethyl-6-methyl- pyrazine	-	-	2.71	-	-	5.21
2-Methyl-5- ethylpyrazine	-	-	3.25	-	-	4.73
Trimethyl pyrazine	6.90	0.40	6.16	0.40	-	11.89
2,6-Diethyl-pyrazine	-	-	1.21	-	-	0.76
2-Ethyl-3,5-dimethyl pyrazine	-	-	6.36	-	-	-
2-Ethyl-3,6-dimethyl- pyrazine	-	-	-	-	-	11.50
2,5-Diethylpyrazine	-	-	-	-	-	0.39
2,5-Diethylpyrazine	-	-	-	-	-	3.23
5-Ethyl-2,3-dimethyl-pyrazine	-	-	1.80	-	-	-
Tetramethyl pyrazine	30.15	1.65	3.87	-	-	-
3,5-Diethyl-2-methyl- pyrazine	-	-	1.12	-	-	-
2,3,5-Ttrimethyl-6- ethylpyrazine	-	-	1.33	-	-	3.10
**subtotal**	**37.05**	**2.05**	**40.71**	**0.4**	**0**	**85.64**
**Others**						
2,3-Dihydrofuran	-	-	2.98	-	-	-
2,5Diethyltetrahydro-furan	1.29	0.25	0.17	-	-	-
2-Acetylfuran	0.75	0.75	3.25	-	-	2.09
Propyl nitrite	4.84	0.93	2.25	-	-	-
2-Acetylpyrrole	-	-	2.08	-	-	-
Benzothiazole	-	-	-	-	-	1.71
Phenol	-	-	-	0.25	-	0.26
**subtotal**	**6.88**	**1.93**	**10.73**	**0.25**	**0.00**	**4.06**

* Each value is expressed as mean ± SD (*n* = 3).

## Data Availability

Not applicable.

## Sample Availability

Samples of the compounds are not available from the authors.
